# Multimodal In‐Sensor Computing with Dual‐Phase Organic Synapses for Wearable Fitness Monitoring

**DOI:** 10.1002/adma.202513904

**Published:** 2025-10-14

**Authors:** Yanran Mao, Yongsuk Choi, Chuan Qian, Dong Gue Roe, Seonkwon Kim, Yuehong Liu, Diandian Chen, Dongsheng Tang, Jia Sun, Jeong Ho Cho

**Affiliations:** ^1^ Key Laboratory of Low‐Dimensional Quantum Structures and Quantum Control of the Ministry of Education Hunan Research Center of the Basic Discipline for Quantum Effects and Quantum Technologies Department of Physics Hunan Normal University Changsha 410081 P. R. China; ^2^ Andrew and Peggy Cherng Department of Medical Engineering California Institute of Technology Pasadena CA 91125 USA; ^3^ Department of Chemical and Biomolecular Engineering Yonsei University Seoul 03722 Republic of Korea; ^4^ Hunan Key Laboratory for Super‐microstructure and Ultrafast Process School of Physics Central South University Changsha 410081 P. R. China

**Keywords:** analog computing, artificial synapse, in‐sensor computing, multi‐modal, wearable device

## Abstract

With the advancement of wearable and mobile devices, demand for the real‐time, low‐power processing of physiological and environmental signals is growing rapidly. To achieve this, neuromorphic systems that employ artificial synapses for analog signal processing and parallel computing represent a promising strategy. In this study, a synaptic sensor is developed that simultaneously responds to human respiration and ambient ultraviolet (UV) light, enabling multimodal analog data processing. The proposed device is fabricated using the organic semiconductor 5,5′‐Di(4‐biphenylyl)‐2,2′‐bithiophene, which has distinct bulk and channel phases. Human respiration‐induced airflow is converted into a synaptic current via charge trapping triggered by the interaction between molecules of water and the bulk phase, leading to real‐time detection of the respiratory rate. The inherent photosensitivity of the device also allows for simultaneous UV detection, thus capturing the environmental exposure conditions. Using these multimodal sensing and processing capabilities, a real‐time feedback system is implemented that supports exercise monitoring by integrating physiological and environmental information. This work demonstrates the potential use of synaptic sensors as front‐end components in wearable neuromorphic platforms, offering a compact, energy‐efficient, and intelligent interface for healthcare and personalized information services.

## Graphical abstract

A dual‐phase organic synaptic sensor detects human respiration and ambient UV light, enabling a wearable neuromorphic system for real‐time exercise monitoring.

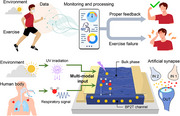

## 1 Introduction

The rapid proliferation of mobile and wearable devices has generated large volumes of data, with the increased interaction between humans and real‐time computing systems driving the demand for personalized information processing.^[^ ^1^, ^2^, ^3^, ^4^, ^5^, ^6^, ^7^, ^8^ ^]^ In order to efficiently handle massive amounts of unstructured data, various brain‐inspired computing technologies, such as artificial intelligence, have been developed.^[^ ^9^, ^10^, ^11^, ^12^, ^13^, ^14^ ^]^ However, current approaches require significant computing resources and an extensive network infrastructure, leading to high power consumption and increasing concerns over privacy and security.^[^ ^13^, ^14^ ^]^ To address these issues, neuromorphic computing, which mimics the data‐processing functions of the brain's neural network using hardware, has received significant attention.^[^ ^15^, ^16^, ^17^, ^18^, ^19^, ^20^, ^21^, ^22^ ^]^ This approach offers real‐time processing and improves energy efficiency via parallel processing based on analog signals, making it a promising candidate for next‐generation massive data processing systems.^[^ ^23^, ^24^, ^25^, ^26^ ^]^

Recent advancements in analog computing technology, including improvements in its cost and energy efficiency, have led to higher demand for new design paradigms suited to increasingly miniaturized, application‐specific devices.^[^ ^27^, ^28^, ^29^, ^30^ ^]^ In particular, artificial synapses capable of implementing various biological synaptic plasticities within a single device have been widely studied,^[^ ^31^, ^32^, ^33^, ^34^, ^35^, ^36^, ^37^, ^38^, ^39^, ^40^, ^41^, ^42^ ^]^ enhancing the functionality and energy efficiency of analog processors.^[^ ^43^, ^44^ ^]^ Research is also underway to develop efficient unit devices that directly combine sensors with artificial synapses for compact bio‐information processing.^[^ ^35^, ^38^, ^45^, ^46^, ^47^, ^48^ ^]^ This integration enables the simultaneous sensing and processing of physiological data, significantly advancing wearable and bio‐interfacing technologies.^[^ ^43^, ^44^, ^48^, ^49^, ^50^ ^]^ By directly handling analog biosignals, synaptic sensors reduce the burden of data transmission and enable efficient, low‐power operation, which are important characteristics for wearable neuromorphic systems.^[^ ^44^, ^45^, ^46^, ^47^ ^]^ The ability to sense and process multiple heterogeneous signals, such as physiological and environmental information, within a single device also represents an important step toward multimodal in‐sensor computing. In particular, it eliminates the need for separate sensor and processor units, allowing compact and energy‐efficient data handling at the device level. These features mean that synaptic sensors have the potential to be vital components of next‐generation neuromorphic systems for wearable healthcare and personalized services.

In this study, we present a synaptic sensor that generates processable analog currents in response to human respiratory activity and ambient ultraviolet (UV) light. The sensor exhibits various forms of synaptic plasticity based on an organic semiconductor channel composed of 5,5′‐Di(4‐biphenylyl)‐2,2′‐bithiophene (BP2T), which has distinct bulk and channel phases. Respiration‐induced airflow is successfully converted into an analog current via the charge trapping triggered by the interaction of H_2_O molecules with the bulk phase of BP2T. The water sensitivity of the synaptic sensor enables the real‐time detection and processing of the respiration rate, which is a key physiological indicator of human health. In addition, due to the photosensitivity of BP2T, UV light can be simultaneously detected, contributing to multimodal data processing via synaptic currents. Based on this multimodal analog processing capability, we demonstrate a real‐time feedback system that supports exercise monitoring by integrating environmental and physiological signals, potentially representing a significant step toward the development of intelligent wearable healthcare systems.

## 2 Results and Discussion

**Figure 1:**
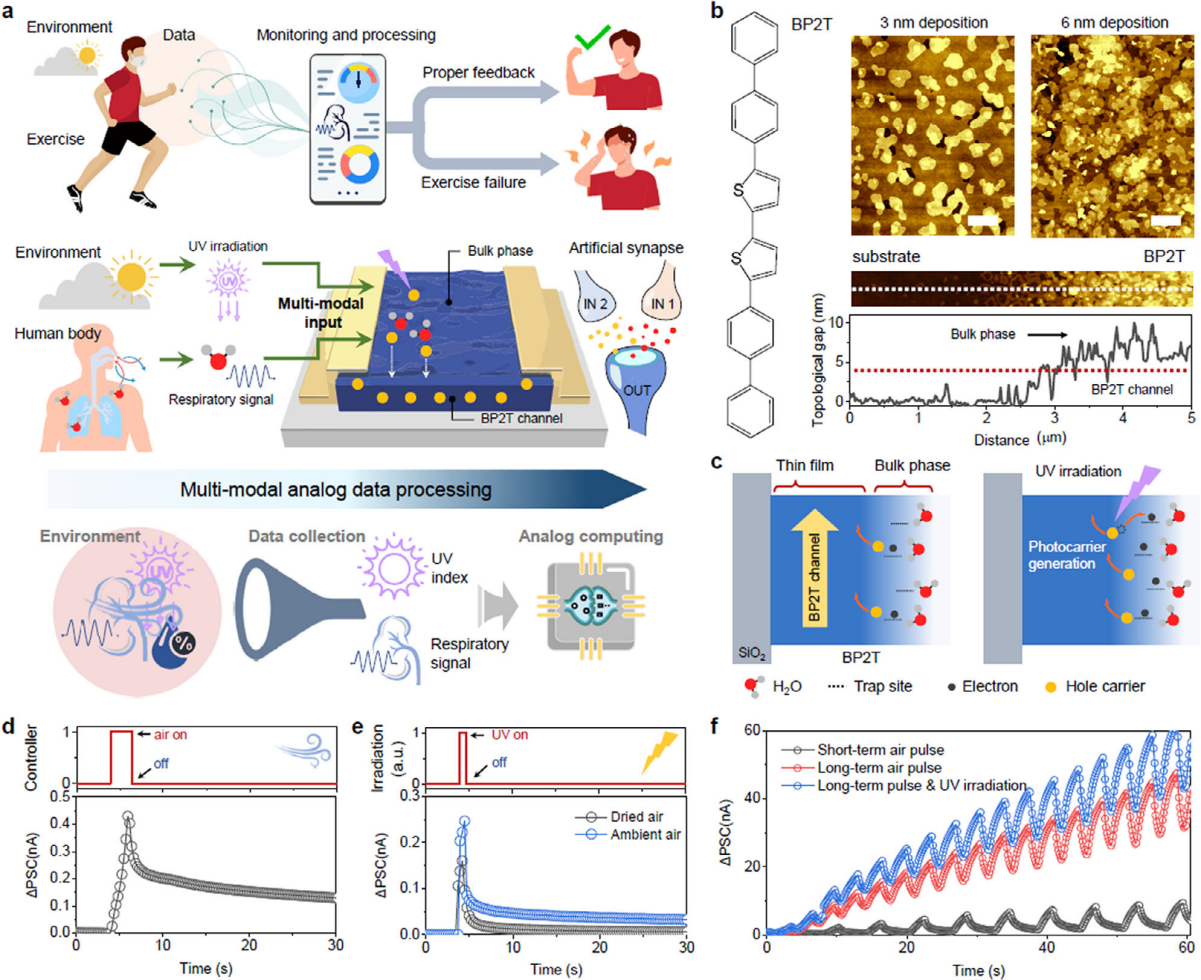
Multi‐modal synaptic sensor for the analog processing of environmental and biomedical data. a) Schematic illustration of a synaptic sensor capable of sensing and processing environmental UV data and biomedical respiratory data simultaneously. b) Molecular structure and morphological characteristics of BP2T. Scale bar, 300 nm c) Schematic illustration of the weight‐changing mechanism for the BP2T synaptic channel. d) Synaptic current response of the BP2T synaptic sensor following exposure to ambient air over a controlled period. e) Synaptic current response to short‐term UV irradiation in dry (black) and ambient air (blue). f) Real‐time change in the synaptic current of the BP2T synaptic sensor exposed to various air and UV irradiation conditions.

When humans engage in exercise, physiological and environmental variables interact to influence the condition of the body. The processing of this physiological and environmental information and subsequent delivery of useful feedback can help minimize the risks associated with exercise and maximize its efficiency. This has increased the demand for wearable devices, communication tools, and expert guidance among both professional athletes and the general public. However, current exercise support systems generally require multiple sensors and significant computational resources to collect and process physiological and environmental data. For this reason, in the present study, we design and test an efficient multimodal data‐processing system based on an artificial synaptic sensor that is capable of simultaneously sensing and processing physiological and environmental information (**Figure** **1**a). BP2T, which is used as the synaptic channel in the proposed device, has a large surface area and exhibits synaptic current modulation in response to both relative humidity (RH) and UV light irradiation, enabling efficient multimodal sensing and analog processing. As a result, our system can directly estimate the respiratory rate and UV intensity based on the synaptic current. This simplifies conventional biomedical data processing by integrating sensing, filtering, and computing into a single device.

Figure 1b presents the molecular structure and deposition characteristics of BP2T. Atomic force microscopy (AFM) imaging revealed two distinctive morphologies during the deposition of BP2T. The initial ≈3 nm of the BP2T was deposited as a continuous film, with further deposition resulting in a bulk‐like structure with a large surface area.^[^ ^51^ ^]^ The continuous film acted as a p‐type semiconducting channel, while the bulk phase provided charge‐trapping sites via the interaction with H_2_O molecules (Figure 1c). In particular, the H_2_O molecules were adsorbed onto the bulk phase, attracting electrons that led to an accumulation of hole carriers in the underlying channel, increasing the p‐channel current. These water traps also captured the electrons generated by BP2T under UV irradiation, suppressing electron–hole recombination and increasing the channel current further.^[^ ^52^ ^]^

The synaptic properties of the BP2T transistor when exposed to air and light stimuli were also evaluated. The BP2T transistor was first placed in a controlled environment and exposed to ambient air (RH = 25%) through a gas valve for 3 s. The probe station chamber was then purged with nitrogen gas. The real‐time synaptic current plot for the BP2T device presented in Figure 1d shows that the post‐synaptic current (PSC) increased when exposed to air and then gradually decreased during purging, indicating that the artificial synapse had excitatory PSC (EPSC) characteristics (V_g_ = 0 V, V_D_ = −10 V). To evaluate the light response of the device, the BP2T artificial synapse device was subsequently exposed to a 340 nm light source (irradiation power of 0.3 W m^−2^), corresponding to the absorption wavelength of BP2T, in both dry and normal air (Figure 1e; Figure S1, Supporting Information). The PSC increased following light stimulation, with the EPSC particularly high when the RH was higher. Figure 1f plots the real‐time synaptic current of the BP2T synaptic sensor when exposed to various air and UV irradiation conditions. The maximum PSC when the device was exposed to a short‐term air pulse cycle consisting of 2 s of ambient air exposure and 4 s of purging was 10 nA after 60 s. However, the PSC increased to 43 nA when the device was exposed to a long‐term air pulse consisting of 4 s of ambient air exposure and 2 s of purging. The PSC increased further to 63 nA when the device was irradiated with UV light (0.3 W m^−2^) together with the air pulses.

**Figure 2:**
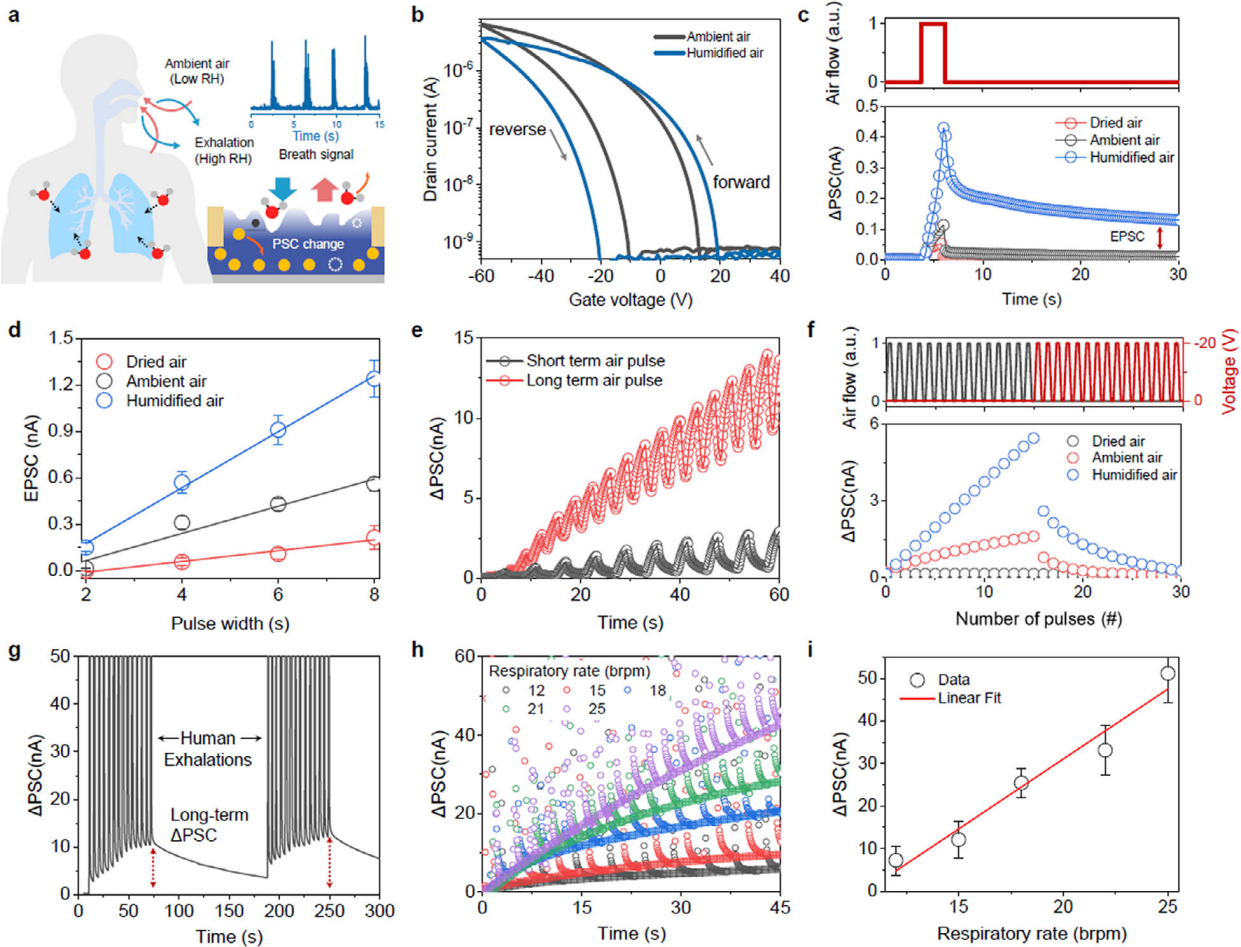
BP2T synaptic sensor designed for processing the human respiratory rate. a) Proof‐of‐concept illustration of a BP2T synapse capable of sensing and processing respiratory data. b) Transfer characteristics of the BP2T synaptic transistor according to the humidity. Measured under consistent UV irradiation of 100 W m^−2^. c) EPSC response of the BP2T synapse under air pulse stimuli with controlled humidity. d) Plots of extracted EPSC values for the BP2T synapse as a function of the air pulse duration. e) Real‐time change in the PSC of the BP2T synaptic sensor exposed to various air conditions. f) Long‐term potentiation (via air pulses) and depression (via voltage pulses) characteristics of the BP2T synapse. g) Real‐time change in the PSC of the BP2T synaptic sensor following repeated exposure to human breathing. h) Change in the PSC of the BP2T synapse as a function of the respiratory rate. i) Calibration plot of extracted ΔPSC values as a function of the respiratory rate. Dots: Average values, Error bars: Standard deviations, n = 5.

Our BP2T synaptic sensor estimated the respiration rate by transducing the periodic change in moisture levels in human respiration into synaptic current (**Figure** **2**a). This is based on the fact that exhaled air has an RH of >80% arising from the air‐exchange process in the lungs. The BP2T synaptic transistor exhibited hysteresis characteristics in the V_G_–I_D_ transfer curve (V_D_ = −40 V, forward V_G_ from 40 to −60 V, reverse V_G_ −60–40 V) due to the presence of various trap sites (Figure 2b). Hysteresis windows of 22 and 39 V were observed in ambient air (RH = 25%) and humidified air (RH = 80%), respectively. The charged trap density was then calculated using Equation (1):

(1) $$N_{trap}=C_{ox}\cdot q^{-1}\cdot \left(VH_{AM}-VH_{HU}\right)$$

where *C*_ox_ is the effective capacitance of the gate dielectric (17 nF·cm^−2^ for the 200 nm SiO_2_ substrate), *VH*_AM_ and *VH*_HU_ are the hysteresis windows in ambient and humid air, respectively, and *q* is the electron charge. Accordingly, it was found that 1.8 × 10^12^ cm^−2^∙eV^−1^ charge trapping sites were generated due to the presence of the H_2_O molecules.

The synaptic properties, which are affected by the trap density, also differed with a change in the RH (Figure 2c). In humid air (RH = 80%), the BP2T synaptic sensor generated an EPSC that was four times higher than that of ambient air (RH = 25%), whereas it was significantly lower when exposed to dry air (RH< 5%). Figure 2d presents the changes in the EPSC as a function of the exposure time for air pulses depending on the RH. The EPSC represents the PSC 20 s after the end of the air pulse input, while the error bar is derived from five repeated measurements. A larger increase in the PSC was observed with a higher frequency of exposure to humid air, suggesting that the H_2_O molecules that remain after purging were adsorbed into the BP2T channels (Figure 2e). On the other hand, the increase in the PSC due to the water‐induced charge traps can be effectively removed using a voltage pulse having a width of 0.1 s, and varied amplitudes (Figure S2, Supporting Information). The response time was less than 0.1 s, ensuring continuous and repeatable analog weight update and reset. As a result, long‐term potentiation and depression (LTP/D) characteristics can be induced in the artificial synapse through a series of air and voltage pulses (Figure 2f).

The performance of the BP2T synaptic sensor in respiratory rate processing was also evaluated with direct exposure to real human breath. Figure 2g presents a real‐time plot of the PSC of the synaptic sensor when exposed to human breathing for 60 s with a resting time of 100 s repeatedly. The PSC rapidly increased due to extremely high RH when exposed to exhalation, before decreasing again during inhalation due to the gradient desorption of water molecules. However, the residual PSC continuously increased during the respiratory cycle due to the influence of the H_2_O molecules adsorbed onto the BP2T surface. Figure 2h presents a real‐time PSC plot for the synaptic sensor with an increase in the respiratory rate from 12 to 25 breaths per minute (brpm). The estimated flow rate of a single breath was 0.25 to 3 L s^−1^, depending on the breath rate. The base current during inhalation for each respiratory cycle is marked with colored lines. For all respiratory rates, the PSC increased due to accumulated H_2_O molecules on the BP2T bulk phase. The base current had a linear correlation with the respiratory rate, indicating that the proposed device successfully operated as a respiratory rate sensor (Figure 2i).

**Figure 3:**
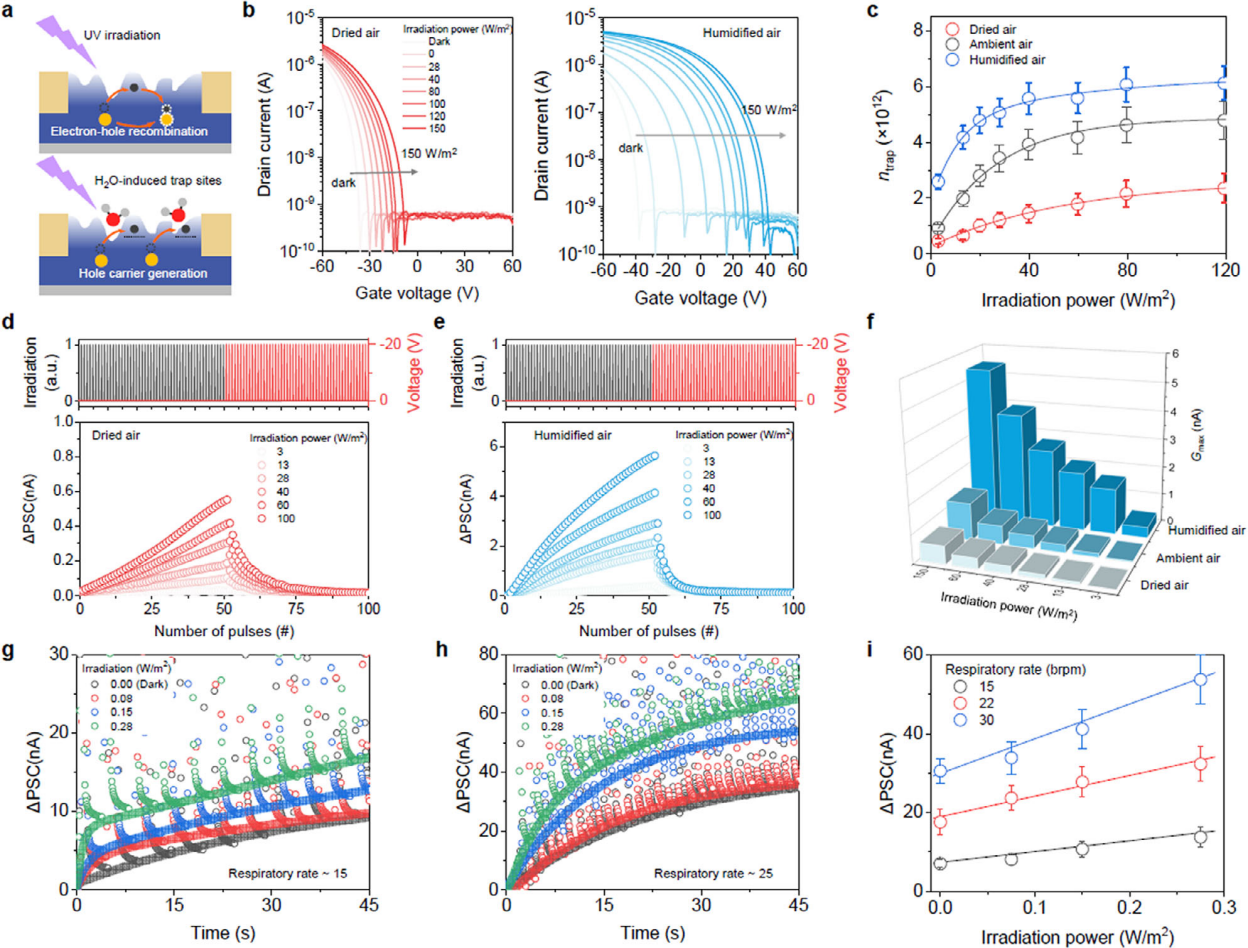
BP2T synaptic sensor designed for the processing of environmental UV irradiation. a) Schematic illustration of the operating mechanisms of the BP2T synapse under UV irradiation. b) Transfer characteristics of the BP2T synaptic transistor following UV irradiation under various humidity conditions. c) Photo‐generated trap density for the BP2T synapse as a function of the irradiation power under various humidity conditions. Dots: Average values, Error bars: Standard deviations, n = 5. Long‐term potentiation (via UV pulses) and depression (via voltage pulses) characteristics of the BP2T synapse were evaluated in d) dry and e) humid air. f) Long‐term potentiation response of the BP2T synapse under various humidity and light irradiation conditions. Change in the PSC of the BP2T synapse in response to human inhalation under various UV irradiation conditions at a respiratory rate of around g) 15 and h) 25 breaths per minute. i) Calibration plot of extracted ΔPSC values as a function of the irradiation power under different respiratory conditions. The dots indicate ΔPSC values extracted 30 s after the breath input. Error bars: Standard deviations, n = 5.

The light‐induced synaptic properties of the BP2T device were also evaluated. Under typical conditions, the electron–hole pairs generated by light irradiation recombine, weakening the electrical characteristics of the device in the short term (**Figure** **3**a). However, when water‐induced trap sites are present in the organic semiconductor channel, photogenerated electrons are captured, preventing the recombination of electron–hole pairs and improving the synaptic properties. Figure 3b presents the transfer characteristics (V_D_ = −40 V) of the BP2T device measured under a UV irradiation power ranging from 0 to 150 W m^−2^ in both dry and humid air (RH = 80%). The device produced a significantly larger increase in the light‐induced current in the humid environment (Figure S3, Supporting Information). The trapped charge calculated based on the threshold shift and hysteresis analysis increased with a higher irradiation power (Figure 3c; Figures S4 and S5, Supporting Information). The change in the charge trap density induced by ambient air was calculated using Equation (2):

(2) $$n_{trap1}=C_{ox}\cdot q^{-1}\cdot \left(VH_{AM}-VH_{D}\right)$$

where *VH*_AM_ and *VH*_D_ are the hysteresis windows for ambient and dry air, respectively (Figure S5, Supporting Information). The change in the charge trap density induced by humidified air was calculated using Equation (3):

(3) $$n_{trap2}=C_{ox}\cdot q^{-1}\cdot \left(VH_{HU}-VH_{D}\right)$$

where *VH*_HU_ is the hysteresis window for the humid air. The overall trap density induced by H_2_O molecules was then determined as *n*_trap2_ − *n*_trap1_, showing that ≈4 × 10^12^ cm^−2^∙eV^−1^ trap sites were consistently generated regardless of the light irradiation power.

The synaptic properties of the BP2T device under light stimulation were also evaluated. As shown in Figure S6 (Supporting Information), the device exhibited EPSC characteristics when irradiated with 340 nm wavelength light. It also displayed stronger and longer‐term memory characteristics because the trap density induced by the H_2_O molecules was higher. LTP/D characteristics were also induced by clearing the trapped charge using voltage pulses (Figure 3d). A larger change in the PSC was observed in the high‐humidity environment, while the LTP/D characteristics were negligible in the dry air (Figure 3e; Figure S7, Supporting Information). Overall, the BP2T synaptic sensor exhibited changes in the PSC in response to both humidity and light stimuli and demonstrated the most potentiated response when H_2_O molecules and light stimuli were present simultaneously (Figure 3f). This synaptic sensory response to light and moisture is a unique characteristic of the BP2T transistor when compared to other organic transistors (Figure S8, Supporting Information). Furthermore, reliability was confirmed through repeated cycle tests (Figure S9, Supporting Information). The device demonstrated stable cycling characteristics within a consistent synaptic weight range during 50 potentiation cycles using light stimuli and humidified air pulses, followed by 50 reset cycles with voltage pulses.

To validate the multimodal signal processing capability of the BP2T synaptic sensor, real human respiration signals were introduced under various light irradiation conditions. Figure S10 (Supporting Information) presents a real‐time PSC plot of the synaptic sensor exposed to breathing stimuli at respiratory rates of 15 and 25 brpm while subjected to different light intensities. The irradiation power was set between 0.0 and 0.3 W m^−^^2^ to simulate typical daily exposure conditions for humans. The PSC recorded for respiration rates of 15 and 25 brpm are shown in Figure 3g,h, respectively, with the base current for each light irradiation level and respiration rate determined using fitting curves. Overall, the PSC of the BP2T synaptic sensor increased linearly with both the light irradiation and respiration rate, confirming its practicality as a sensor and processing element (Figure 3i). Furthermore, the clear enhancement of the PSC and the greater slope change observed under high‐humidity conditions signify a synergistic effect between light and humidity. This is due to a physical coupling mechanism in which water‐induced traps capture the photo‐generated charge carriers, leading to a non‐linear amplification of the photocurrent.^[^ ^52^ ^]^

**Figure 4:**
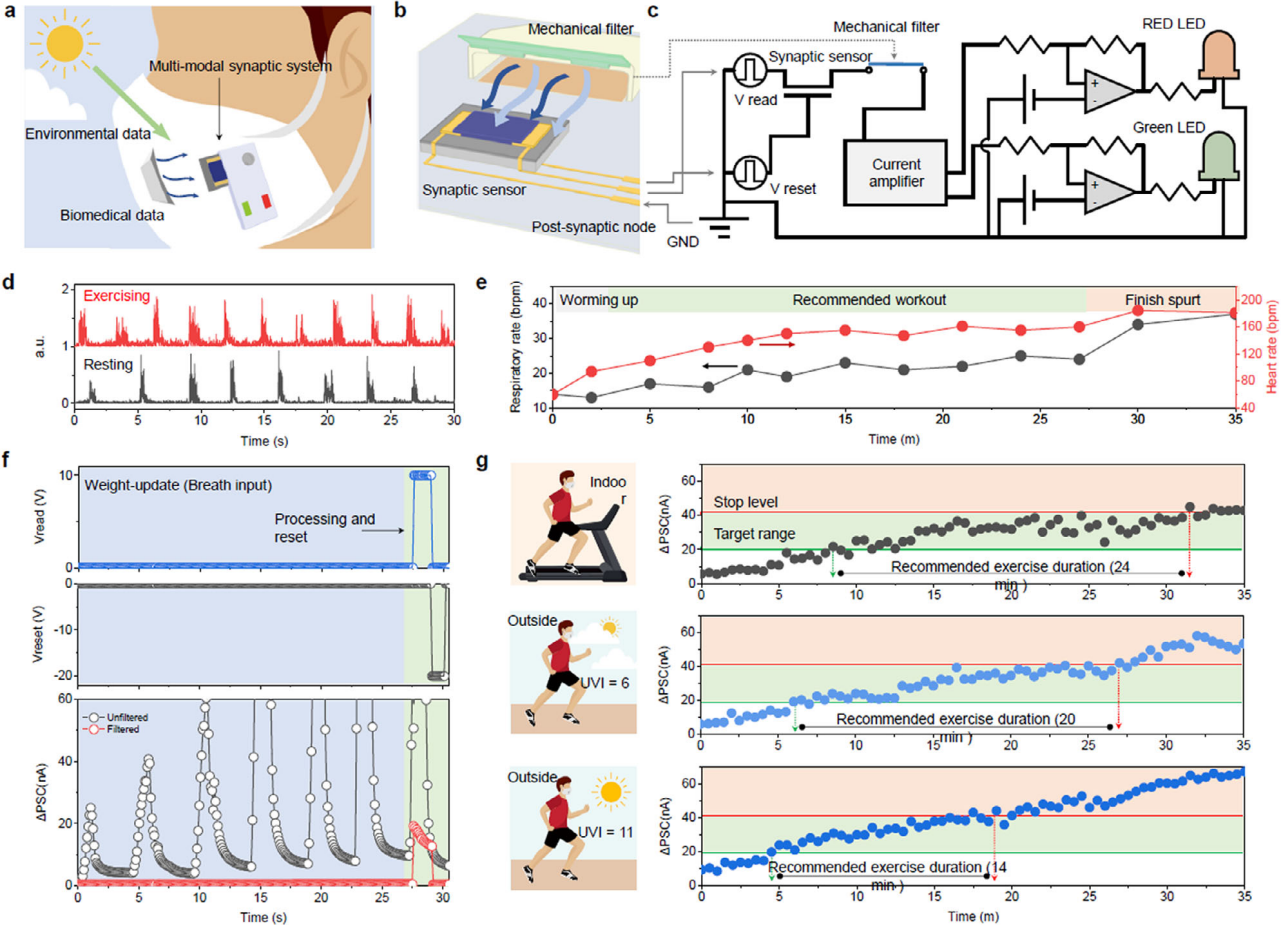
Evaluation of the BP2T synaptic sensor in a simulated wearable exercise support system. a) Proof‐of‐concept illustration of a wearable exercise support system utilizing the proposed BP2T synaptic sensor. b) Schematic illustration of respiratory information processing using the BP2T synaptic sensor. c) Simplified circuit diagram of the exercise support system. d) Human respiratory signals under normal conditions (black) and during exercise (red). In normal conditions, sit down and rest for 10 min. Exercise, ride an indoor cycle at moderate intensity for 20 min. e) Changes in the heart rate and respiratory rate during 35 min of exercise. f) Read voltage and reset voltage settings used to operate the exercise support system, and changes in the PSC observed during respiratory signal processing. g) Change in the PSC during 35 min of exercise under various environmental conditions.

A multimodal analog signal processing system was also developed to assist human exercise. **Figure** **4**a presents the conceptual design of this analog exercise‐support system. The BP2T synaptic sensor was integrated into a virtual mask, where it detected UV signals from the external environment. Simultaneously, it captured exhalation signals through a physical filter embedded in the mask (Figure 4b). The synaptic sensor was connected to a current amplifier and output terminals that included a comparator circuit and an LED indicator. This indicator reflected whether the exercise intensity was within an appropriate range (green) or whether it should be reduced (red) based on the analog signal derived from the combined UV and respiratory inputs. A detailed schematic of the overall system circuit is shown in Figure 4c. The comparator connected to the green LED was set with a corresponding threshold current of 20 nA, while the red LED was activated when the PSC exceeded 40 nA (Figure S11, Supporting Information). These threshold values were determined based on the calibration data. The human study and breath collection were designed to strictly adhere to established safety regulations.

To validate this system, we simulated exercise scenarios involving the human body in indoor and outdoor conditions. In general, adults exhibit a resting respiratory rate of 15–20 brpm, which increases to 20–30 brpm during physical activity (Figure 4d). While more intense exercise can lead to breathing rates over 35, the oxygen exchange efficiency declines beyond a certain threshold, making exercise in this range inadvisable.^[^ ^53^ ^]^ Figure 4e presents the respiratory rate and heart rate of a human subject who exercised for a total duration of 35 min. The heart rate was used as a general indicator to determine the exercise status. The subject maintained the recommended exercise intensity for ≈30 min, followed by a 5 min period of increased intensity until exhaustion. During the recommended exercise, both the respiratory rate and heart rate gradually increased over time, with a rapid rise recorded during the final sprint phase. During this exercise, the exposure of the BP2T synaptic sensor to UV occurred within a probe station chamber, while the subject's breath was delivered through a controlled pipeline. Complete details of the operation of this system are presented in Figure 4f. Data processing occurred within a total duration of 30 s, consisting of ≈27 s of respiration updating under light irradiation, followed by 1.5 s for the PSC current readout and 1.5 s for the PSC reset via a weight control voltage. The estimated power consumption for this operation ranged from 6 to 34 nW, which is highly competitive with recently reported synaptic sensors for healthcare applications.^[^ ^54^ ^]^ During the PSC readout, exposure to exhalation was prevented by a mechanical filter to ensure measurement stability. Furthermore, the effect of unintended interference, i.e., humidity changes and pressure variance from unintended wind effects, was evaluated. As shown in Figure S12 (Supporting Information), the device was exposed to artificially generated fan wind along with human breath for 30 s. The effects of the wind and ambient air humidity were negligible, with the change in PSC dominated by the respiration.

Our system was tested under simulated conditions representing an indoor environment, an outdoor environment during the day (UV index = 6), and an outdoor environment under intense UV radiation (UV index = 11) (Figure 4g). In the indoor exercise environment, the red LED was activated at ≈32 min, coinciding with the failure point shown in Figure 4e. The duration of appropriate exercise—defined as the period during which only the green LED was on—was calculated to be ≈24 min. Under outdoor conditions with UV exposure, the red LED activated earlier, resulting in a shorter recommended exercise interval. In particular, in the environment with a UV index of 11, the recommended exercise time was reduced to 14 min, representing a 40% decrease compared to the indoor exercise. This reduction is in accordance with exercise duration guidelines in UV environments recommended by various health organizations.^[^ ^53^, ^55^ ^]^ These results demonstrate that the BP2T synaptic sensor can accurately process both human activity and environmental data, thus supporting complex healthcare functionalities such as exercise assistance. The compact, multi‐modal, and analog nature of our device promises a new paradigm for efficient, intelligent, and scalable wearable neuromorphic systems, outperforming conventional single‐function sensors.

## 3 Conclusion

In summary, we demonstrated a synaptic sensor that generated an analog current in response to human respiration and ambient UV light. The device exhibited excitatory synaptic plasticity based on the fabrication of an organic semiconductor channel based on BP2T with distinct bulk and channel phases. Respiration‐induced airflow was converted into analog signals via charge trapping triggered by the interaction of H_2_O molecules with the bulk phase, enabling the real‐time processing of the respiratory rate. The photosensitivity of BP2T also allowed the simultaneous detection of UV, supporting multimodal data processing. Based on these capabilities, we demonstrated a real‐time feedback system that monitored exercise intensity by integrating physiological and environmental signals. Overall, the initial threshold‐based computing demonstrated in this study lays the foundation for more sophisticated in‐sensor processing. By extending the system to include multi‐threshold detection, extensive biomarkers, and further dynamic learning mechanisms, our device could enable a new class of intelligent, adaptive wearable healthcare devices capable of refined responses to a variety of physiological and environmental signals. These findings highlight the potential of synaptic sensors for use in next‐generation wearable neuromorphic systems and personalized healthcare applications.

## 4 Experimental Section

The sensor device was fabricated on a 200 nm SiO_2_/Si substrate. The substrate was initially ultrasonicated in ethanol and deionized water in sequence, followed by drying under a flow of N_2_ and ozone treatment. BP2T purchased from Sigma–Aldrich was fabricated as a thin film using thermal evaporation through a shadow mask with a deposition rate of 0.2–0.3 Å s^−1^ and a substrate temperature of 80 °C. A 60 nm‐thick Au layer was then deposited using electron beam evaporation through a shadow mask to produce a channel with a length of 50 µm and a width of 1000 µm. The artificial sensory synapses were measured using a Keithley 4200 semiconductor parameter analyzer in a Lake Shore test chamber. A UV laser was used as the light source, with the light power adjusted by varying the input voltage and calibrated using an optical power meter (Sanwa LP10) placed in the same position as the device. Gas pulses were generated using an intermittent pump. The RH in the test chamber was calibrated using a hygrometer. The surface morphology and thickness of the thin films were characterized using atomic force microscopy (AFM, Bruker Dimension ICON). Study participants provided written informed consent prior to their involvement in the research. The evaluation of the BP2T synaptic sensor was conducted in human subjects and strictly adheres to established ethical guidelines as delineated in protocols approved by the Institutional Review Board (IRB) at Hunan Normal University (#619).

## Conflict of Interest

The authors declare no competing financial interest.

## Supporting information



Supporting Information

## Data Availability

The data that support the findings of this study are available from the corresponding author upon reasonable request.

## References

[1] J. Shin , J. W. Song , M. T. Flavin , S. Cho , S. Li , A. Tan , K. R. Pyun , A. G. Huang , H. Wang , S. Jeong , K. E. Madsen , J. Trueb , M. Kim , K. Nguyen , A. Yang , Y. Hsu , W. Sung , J. Lee , S. Phyo , J.‐H. Kim , A. Banks , J.‐K. Chang , A. S. Paller , Y. Huang , G. A. Ameer , J. A. Rogers , Nature 2025, 640, 375. 40205217 10.1038/s41586-025-08825-2 PMC12182646

[2] K. Kwon , J. U. Kim , Y. Deng , S. R. Krishnan , J. Choi , H. Jang , K. Lee , C.‐J. Su , I. Yoo , Y. Wu , L. Lipschultz , J.‐H. Kim , T. S. Chung , D. Wu , Y. Park , T. Kim , R. Ghaffari , S. Lee , Y. Huang , J. A. Rogers , Nat. Electron. 2021, 4, 302.

[3] N. Brasier , J. Wang , W. Gao , J. R. Sempionatto , C. Dincer , H. C. Ates , F. Güder , S. Olenik , I. Schauwecker , D. Schaffarczyk , E. Vayena , N. Ritz , M. Weisser , S. Mtenga , R. Ghaffari , J. A. Rogers , J. Goldhahn , Nature 2024, 636, 57. 39633192 10.1038/s41586-024-08249-4 PMC12007731

[4] H. C. Ates , P. Q. Nguyen , L. Gonzalez‐Macia , E. Morales‐Narváez , F. Güder , J. J. Collins , C. Dincer , Nat. Rev. Mater. 2022, 7, 887. 35910814 10.1038/s41578-022-00460-x PMC9306444

[5] X. Chen , D.‐H. Kim , N. Lu , Chem. Rev. 2024, 124, 6145. 38773952 10.1021/acs.chemrev.4c00271

[6] M. Tan , Y. Xu , Z. Gao , T. Yuan , Q. Liu , R. Yang , B. Zhang , L. Peng , Adv. Mater. 2022, 34, 2108491. 10.1002/adma.202108491 35008128

[7] L. Kong , W. Li , T. Zhang , H. Ma , Y. Cao , K. Wang , Y. Zhou , A. Shamim , L. Zheng , X. Wang , W. Huang , Adv. Mater. 2024, 36, 2400333. 10.1002/adma.202400333 38652082

[8] Y. Lin , M. Bariya , A. Javey , Adv. Funct. Mater. 2021, 31, 2008087.

[9] A. Mehonic , A. J. Kenyon , Nature 2022, 604, 255. 35418630 10.1038/s41586-021-04362-w

[10] G. Li , L. Deng , H. Tang , G. Pan , Y. Tian , K. Roy , W. Maass , Proc. IEEE 2024, 112, 544.

[11] T. J. Park , S. Deng , S. Manna , A. N. M. N. Islam , H. Yu , Y. Yuan , D. D. Fong , A. A. Chubykin , A. Sengupta , S. K. R. S. Sankaranarayanan , S. Ramanathan , Adv. Mater. 2023, 35, 2203352. 10.1002/adma.202203352 35723973

[12] L. G. Wright , T. Onodera , M. M. Stein , T. Wang , D. T. Schachter , Z. Hu , P. L. McMahon , Nature 2022, 601, 549. 35082422 10.1038/s41586-021-04223-6 PMC8791835

[13] H. Wang , T. Fu , Y. Du , W. Gao , K. Huang , Z. Liu , P. Chandak , S. Liu , P. Van Katwyk , A. Deac , A. Anandkumar , K. Bergen , C. P. Gomes , S. Ho , P. Kohli , J. Lasenby , J. Leskovec , T.‐Y. Liu , A. Manrai , D. Marks , B. Ramsundar , L. Song , J. Sun , J. Tang , P. Veličković , M. Welling , L. Zhang , C. W. Coley , Y. Bengio , M. Zitnik , Nature 2023, 620, 47. 37532811 10.1038/s41586-023-06221-2

[14] M. Krenn , R. Pollice , S. Y. Guo , M. Aldeghi , A. Cervera‐Lierta , P. Friederich , G. dos Passos Gomes , F. Häse , A. Jinich , A. Nigam , Z. Yao , A. Aspuru‐Guzik , Nat. Rev. Phys. 2022, 4, 761. 36247217 10.1038/s42254-022-00518-3 PMC9552145

[15] L. Tong , Z. Peng , R. Lin , Z. Li , Y. Wang , X. Huang , K.‐H. Xue , H. Xu , F. Liu , H. Xia , P. Wang , M. Xu , W. Xiong , W. Hu , J. Xu , X. Zhang , L. Ye , X. Miao , Science 2021, 373, 1353. 34413170 10.1126/science.abg3161

[16] D. V. Christensen , R. Dittmann , B. Linares‐Barranco , A. Sebastian , M. L.e Gallo , A. Redaelli , S. Slesazeck , T. Mikolajick , S. Spiga , S. Menzel , I. Valov , G. Milano , C. Ricciardi , S.‐J. Liang , F. Miao , M. Lanza , T. J. Quill , S. T. Keene , A. Salleo , J. Grollier , D. Marković , A. Mizrahi , P. Yao , J. J. Yang , G. Indiveri , J. P. Strachan , S. Datta , E. Vianello , A. Valentian , J. Feldmann , et al., Neuromorphic Comput. Eng. 2022, 2, 022501.

[17] W. Zhang , P. Yao , B. Gao , Q. Liu , D. Wu , Q. Zhang , Y. Li , Q. Qin , J. Li , Z. Zhu , Y. Cai , D. Wu , J. Tang , H. Qian , Y. Wang , H. Wu , Science 2023, 381, 1205. 37708281 10.1126/science.ade3483

[18] K.‐U. Demasius , A. Kirschen , S. Parkin , Nat. Electron. 2021, 4, 748.

[19] H. Lin , J. Ou , Z. Fan , X. Yan , W. Hu , B. Cui , J. Xu , W. Li , Z. Chen , B. Yang , K. Liu , L. Mo , M. Li , X. Lu , G. Zhou , X. Gao , J.‐M. Liu , Nat. Commun. 2025, 16, 421. 39774072 10.1038/s41467-024-55508-z PMC11707328

[20] T. Xiong , C. Li , X. He , B. Xie , J. Zong , Y. Jiang , W. Ma , F. Wu , J. Fei , P. Yu , L. Mao , Science 2023, 379, 156. 36634194 10.1126/science.adc9150

[21] D. Marković , A. Mizrahi , D. Querlioz , J. Grollier , Nat. Rev. Phys. 2020, 2, 499.

[22] K. Roy , A. Jaiswal , P. Panda , Nature 2019, 575, 607. 31776490 10.1038/s41586-019-1677-2

[23] X. Mou , J. Tang , Y. Lyu , Q. Zhang , S. Yang , F. Xu , W. Liu , M. Xu , Y. Zhou , W. Sun , Y. Zhong , B. Gao , P. Yu , H. Qian , H. Wu , Sci. Adv. 2021, 7, abh0648. 10.1126/sciadv.abh0648 PMC8284889 34272239

[24] E. J. Fuller , S. T. Keene , A. Melianas , Z. Wang , S. Agarwal , Y. Li , Y. Tuchman , C. D. James , M. J. Marinella , J. J. Yang , A. Salleo , A. A. Talin , Science 2019, 364, 570. 31023890 10.1126/science.aaw5581

[25] D. Kwon , S. Y. Woo , K.‐H. Lee , J. Hwang , H. Kim , S.‐H. Park , W. Shin , J.‐H. Bae , J.‐J. Kim , J.‐H. Lee , Sci. Adv. 2023, 9, adg9123. 10.1126/sciadv.adg9123 PMC10355823 37467329

[26] C. Wang , S.‐J. Liang , C.‐Y. Wang , Z.‐Z. Yang , Y. Ge , C. Pan , X. Shen , W. Wei , Y. Zhao , Z. Zhang , B. Cheng , C. Zhang , F. Miao , Nat. Nanotechnol. 2021, 16, 1079. 34239120 10.1038/s41565-021-00943-y

[27] X. Zhao , H. Zou , M. Wang , J. Wang , T. Wang , L. Wang , X. Chen , Adv. Mater. 2024, 36, 2403444. 10.1002/adma.202403444 38934554

[28] A. S. Goossens , M. Ahmadi , D. Gupta , I. Bhaduri , B. J. Kooi , T. Banerjee , Adv. Electron. Mater. 2023, 9, 2201111.

[29] V. K. Sangwan , M. C. Hersam , Nat. Nanotechnol. 2020, 15, 517. 32123381 10.1038/s41565-020-0647-z

[30] X. Yan , J. H. Qian , V. K. Sangwan , M. C. Hersam , Adv. Mater. 2022, 34, 2108025. 10.1002/adma.202108025 34813677

[31] Y. Kim , A. Chortos , W. Xu , Y. Liu , J. Y. Oh , D. Son , J. Kang , A. M. Foudeh , C. Zhu , Y. Lee , S. Niu , J. Liu , R. Pfattner , Z. Bao , T.‐W. Lee , Science 2018, 360, 998. 29853682 10.1126/science.aao0098

[32] S. Chen , T. Zhang , S. Tappertzhofen , Y. Yang , I. Valov , Adv. Mater. 2023, 35, 2301924. 10.1002/adma.202301924 37199224

[33] F. Molina‐Lopez , T. Z. Gao , U. Kraft , C. Zhu , T. Öhlund , R. Pfattner , V. R. Feig , Y. Kim , S. Wang , Y. Yun , Z. Bao , Nat. Commun. 2019, 10, 2676. 31213599 10.1038/s41467-019-10569-3 PMC6582140

[34] H. Wei , R. Shi , L. Sun , H. Yu , J. Gong , C. Liu , Z. Xu , Y. Ni , J. Xu , W. Xu , Nat. Commun. 2021, 12, 1068. 33594066 10.1038/s41467-021-21319-9 PMC7886898

[35] W. Wang , Y. Jiang , D. Zhong , Z. Zhang , S. Choudhury , J.‐C. Lai , H. Gong , S. Niu , X. Yan , Y. Zheng , C.‐C. Shih , R. Ning , Q. Lin , D. Li , Y.‐H. Kim , J. Kim , Y.‐X. Wang , C. Zhao , C. Xu , X. Ji , Y. Nishio , H. Lyu , J. B.‐H. Tok , Z. Bao , Science 2023, 380, 735. 37200416 10.1126/science.ade0086

[36] H. Wei , H. Yu , J. Gong , M. Ma , H. Han , Y. Ni , S. Zhang , W. Xu , Adv. Funct. Mater. 2021, 31, 2007232.

[37] H. Bian , Y. Y. Goh , Y. Liu , H. Ling , L. Xie , X. Liu , Adv. Mater. 2021, 33, 2006469. 10.1002/adma.202006469 33837601

[38] Y. Lee , J. Y. Oh , W. Xu , O. Kim , T. R. Kim , J. Kang , Y. Kim , D. Son , J. B.‐H. Tok , M. J. Park , Z. Bao , T.‐W. Lee , Sci. Adv. 2018, 4, aat7387. 10.1126/sciadv.aat7387 PMC6251720 30480091

[39] S. T. Keene , C. Lubrano , S. Kazemzadeh , A. Melianas , Y. Tuchman , G. Polino , P. Scognamiglio , L. Cinà , A. Salleo , Y. van de Burgt , F. Santoro , Nat. Mater. 2020, 19, 969. 32541935 10.1038/s41563-020-0703-y

[40] T. Wang , M. Wang , J. Wang , L. Yang , X. Ren , G. Song , S. Chen , Y. Yuan , R. Liu , L. Pan , Z. Li , W. R. Leow , Y. Luo , S. Ji , Z. Cui , K. He , F. Zhang , F. Lv , Y. Tian , K. Cai , B. Yang , J. Niu , H. Zou , S. Liu , G. Xu , X. Fan , B. Hu , X. J. Loh , L. Wang , X. Chen , Nat. Electron. 2022, 5, 586.

[41] F. Wu , P. Yu , L. Mao , Innov. Mater. 2023, 1, 100007.

[42] P. C. Harikesh , D. Tu , S. Fabiano , Nat. Electron. 2024, 7, 525.

[43] D. Chen , Y. Choi , C. Qian , D. G. Roe , H. Kim , S. B. Jo , Y. Yoo , D. Tang , J. H. Cho , Adv. Funct. Mater. 2024, 34, 2405244.

[44] E. Covi , E. Donati , X. Liang , D. Kappel , H. Heidari , M. Payvand , W. Wang , Front. Neurosci. 2021, 15, 2021. 10.3389/fnins.2021.611300 PMC8144334 34045939

[45] T. Sun , B. Feng , J. Huo , Y. Xiao , W. Wang , J. Peng , Z. Li , C. Du , W. Wang , G. Zou , L. Liu , Nano‐Micro Lett. 2023, 16, 14. 10.1007/s40820-023-01235-x PMC10643743 37955844

[46] F. Torricelli , D. Z. Adrahtas , Z. Bao , M. Berggren , F. Biscarini , A. Bonfiglio , C. A. Bortolotti , C. D. Frisbie , E. Macchia , G. G. Malliaras , I. McCulloch , M. Moser , T.‐Q. Nguyen , R. M. Owens , A. Salleo , A. Spanu , L. Torsi , Nat. Rev. Methods Primer 2021, 1, 66. 10.1038/s43586-021-00065-8 PMC9037952 35475166

[47] I. Krauhausen , D. A. Koutsouras , A. Melianas , S. T. Keene , K. Lieberth , H. Ledanseur , R. Sheelamanthula , A. Giovannitti , F. Torricelli , I. Mcculloch , P. W. M. Blom , A. Salleo , Y. van de Burgt , P. Gkoupidenis , Sci. Adv. 2021, 7, abl5068. 10.1126/sciadv.abl5068 PMC8664264 34890232

[48] Y. Choi , D. H. Ho , S. Kim , Y. J. Choi , D. G. Roe , I. C. Kwak , J. Min , H. Han , W. Gao , J. H. Cho , Sci. Adv. 2023, 9, adg5946. 10.1126/sciadv.adg5946 PMC10321737 37406117

[49] L. Chen , M. Ren , J. Zhou , X. Zhou , F. Liu , J. Di , P. Xue , C. Li , Q. Li , Y. Li , L. Wei , Q. Zhang , Proc. Natl. Acad. Sci 2024, 121, 2407971121. 10.1073/pnas.2407971121 PMC11331142 39110725

[50] U. Jung , M. Kim , J. Jang , J.‐H. Bae , I. M. Kang , S.‐H. Lee , Adv. Sci. 2024, 11, 2307494. 10.1002/advs.202307494 PMC10916635 38087893

[51] L. Huang , C. Liu , B. Yu , J. Zhang , Y. Geng , D. Yan , J. Phys. Chem. B 2010, 114, 4821.

[52] R. Jia , X. Wu , W. Deng , X. Zhang , L. Huang , K. Niu , L. Chi , J. Jie , Adv. Funct. Mater. 2019, 29, 1905657.

[53] T. J. Wetter , C. M. St Croix , D. F. Pegelow , D. A. Sonetti , J. A. Dempsey , J. Appl. Physiol. 2001, 91, 847. 11457802 10.1152/jappl.2001.91.2.847

[54] Y. Choi , P. Jin , S. Lee , Y. Song , R. Y. Tay , G. Kim , J. Yoo , H. Han , J. Yeom , J. H. Cho , D.‐H. Kim , W. Gao , Nat. Commun. 2025, 16, 5689. 40593594 10.1038/s41467-025-60854-7 PMC12216833

[55] A. Snyder , M. Valdebran , D. Terrero , K. T. Amber , K. M. Kelly , Sports Med. – Open 2020, 6, 42. 32880767 10.1186/s40798-020-00272-9 PMC7471243

