# Supplementary material for: Multimodal In‐Sensor Computing with Dual‐Phase Organic Synapses for Wearable Fitness Monitoring
Source: Adv Mater. 2025 Oct 14;38(4):e13904. doi: 10.1002/adma.202513904 (PMC12810669; doi:10.1002/adma.202513904)
Supplement: Supplementary file 1 — Supporting Information [file ADMA-38-e13904-s001.docx]

Supporting Information for

**Multimodal In-Sensor Computing with Dual-Phase Organic Synapses for Wearable Fitness Monitoring**

Yanran Mao,^1,†^ Yongsuk Choi,^2,†^ Chuan Qian,^1,*^ Dong Gue Roe,^3^ Seonkwon Kim^3^, Yuehong Liu,^1^ Diandian Chen,^1^ Dongsheng Tang,^1^ Jia Sun,^4,*^ and Jeong Ho Cho^3,*^

^1^Key Laboratory of Low-Dimensional Quantum Structures and Quantum Control of the Ministry of Education, Hunan Research Center of the Basic Discipline for Quantum Effects and Quantum Technologies, Department of Physics, Hunan Normal University, Changsha, 410081, P.R. China.

^2^Andrew and Peggy Cherng Department of Medical Engineering, California Institute of Technology, Pasadena, CA 91125, USA.

^3^Department of Chemical and Biomolecular Engineering, Yonsei University, Seoul 120-749, Republic of Korea.

^4^Hunan Key Laboratory for Super-microstructure and Ultrafast Process, School of Physics, Central South University, Changsha, 410081, P.R. China.

^†^Y. Mao and Y. Choi contributed equally to this work.

*Corresponding author: C. Qian ([qianchuan@hunnu.edu.cn](mailto:qianchuan@hunnu.edu.cn)); J. Sun (jiasun@csu.edu.cn); J. H. Cho ([jhcho94@yonsei.ac.kr](mailto:jhcho94@yonsei.ac.kr))


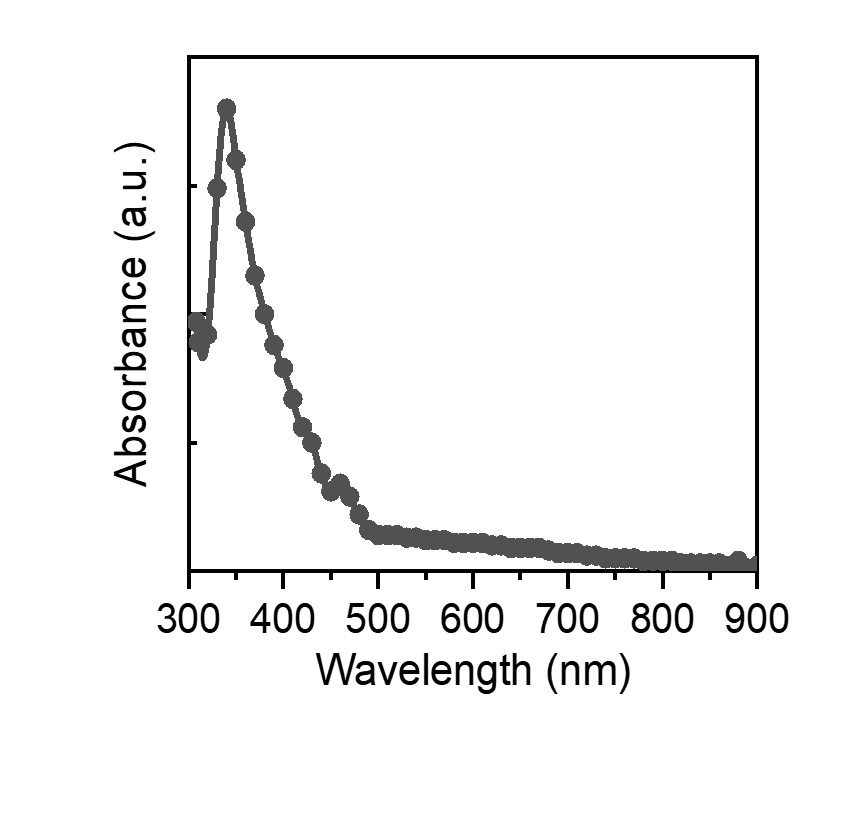


**Fig. S1 |** Absorbance of BP2T using ultraviolet–visible spectroscopy.


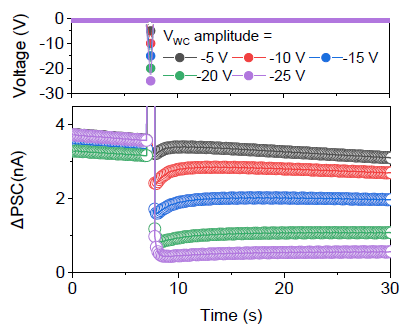


**Fig. S2 |** Inhibitory post-synaptic current (IPSC) of the BP2T synapse according to the depression voltage. Pulse width, 0.1 s.


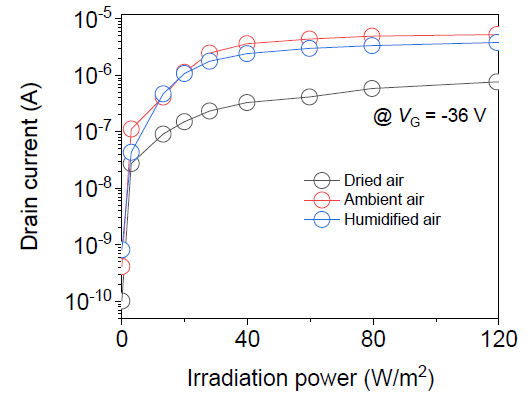


**Fig. S3 |** Drain current of the BP2T transistor at a fixed gate voltage under various humidity and irradiation conditions.


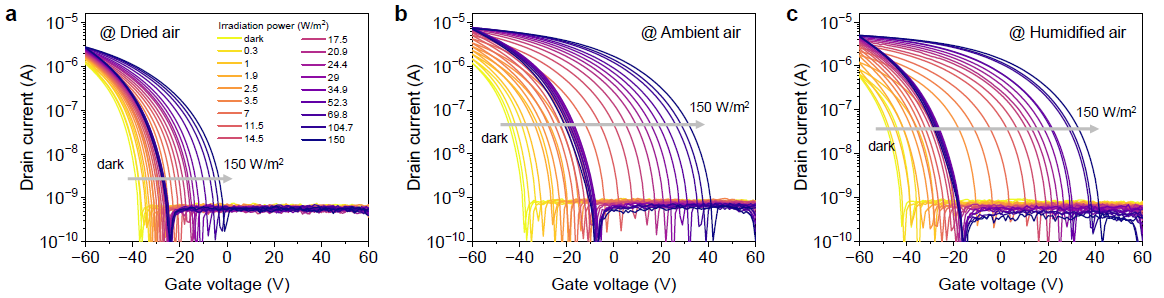


**Fig. S4 |** Transfer curves for the BP2T transistor under various irradiation conditions in **a**, dry, **b**, ambient, and **c**, humid air.


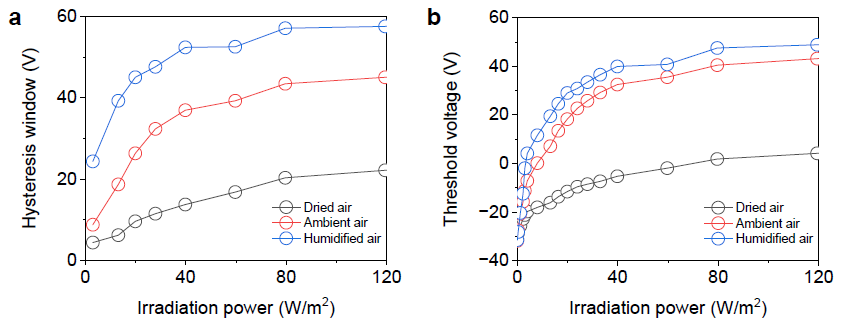


**Fig. S5 | a**, Hysteresis windows and **b**, threshold voltage for the BP2T transistor under various irradiation and humidity conditions.


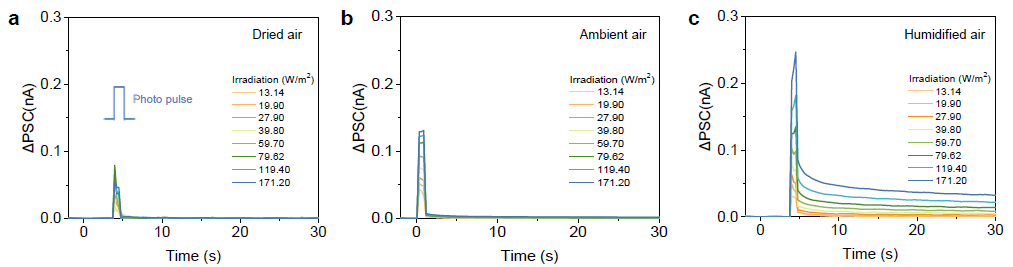


**Fig. S6 |** EPSC characteristics of the BP2T synaptic sensor under various irradiation conditions in **a**, dry, **b**, ambient, and **c**, humid air.


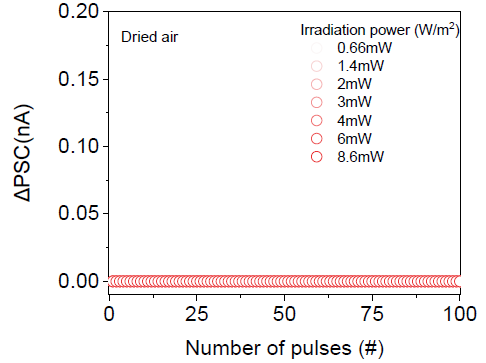


**Fig. S7 |** LTP/D characteristics of the BP2T synaptic sensor in dry air.


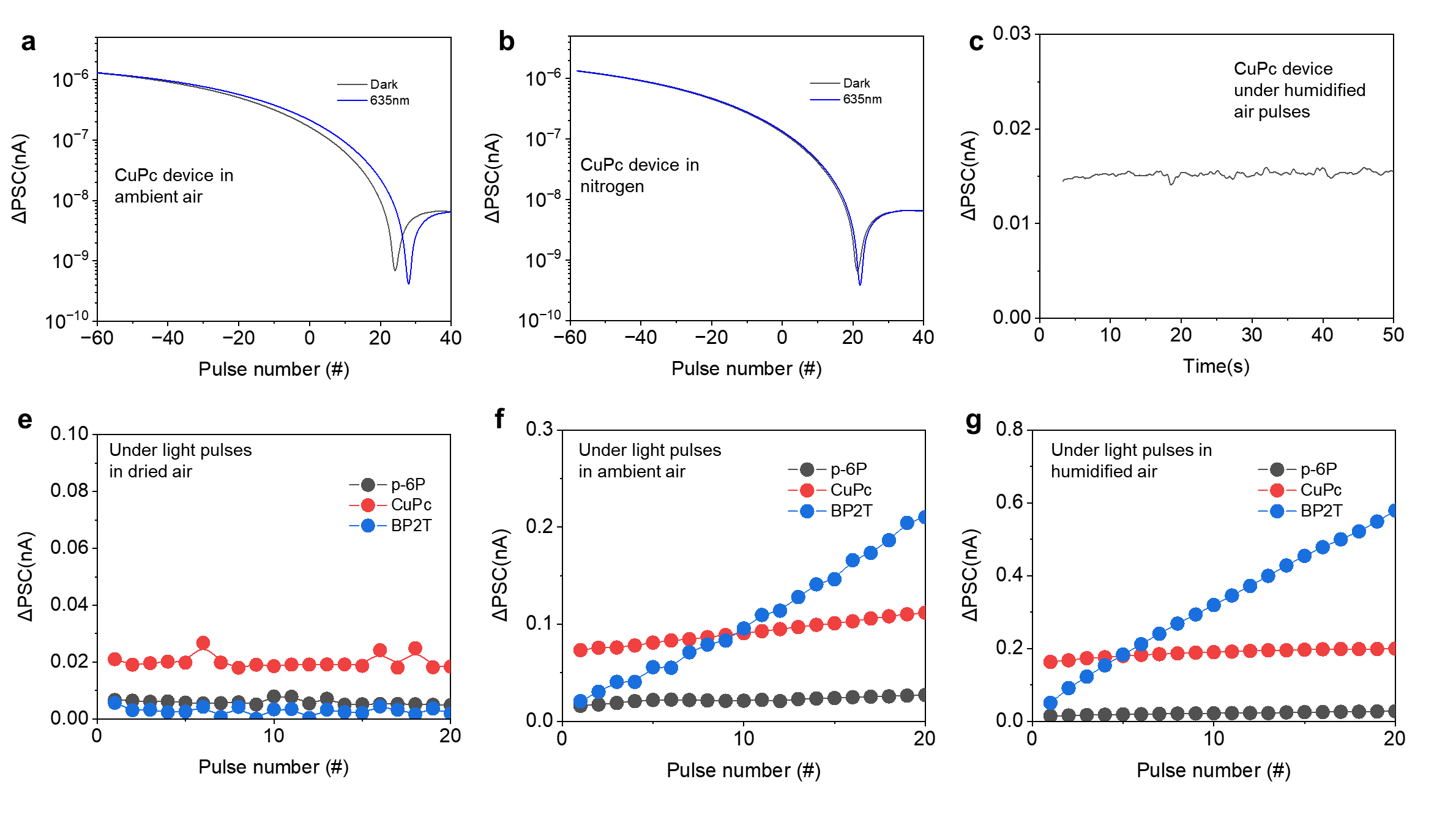


**Fig. S8 |** Transfer curves for the CuPc transistor under exposure of active wavelength light in **a**, ambient air, and **b**, nitrogen. **c**, EPSC response of the CuPc device under humidified air pulse stimuli. Long-term potentiation characteristics of p-6P, CuPc, and BP2T synaptic devices under light pulses in **e**, dry, **f**, ambient, and **g**, humid air.


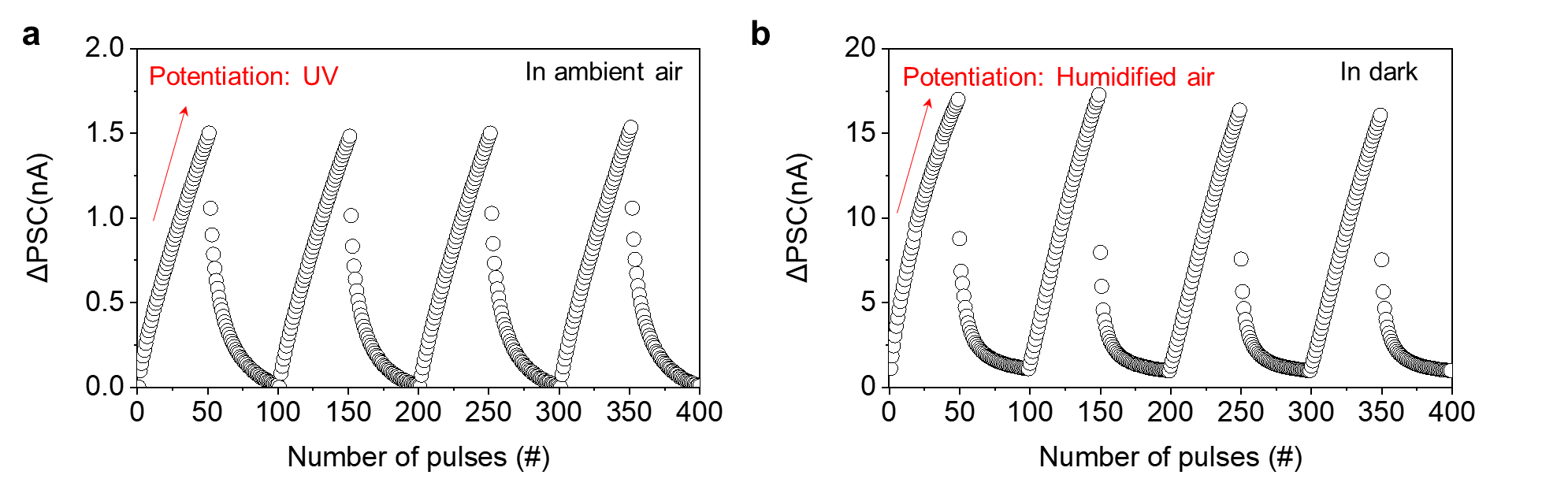


**Fig. S9 |** LTP/D characteristics in four repeated potentiation/depression cycles. **a**, Potentiation with UV light while depressing with voltage pulses. **b**, Potentiation with air pulses while depressing with voltage pulses.


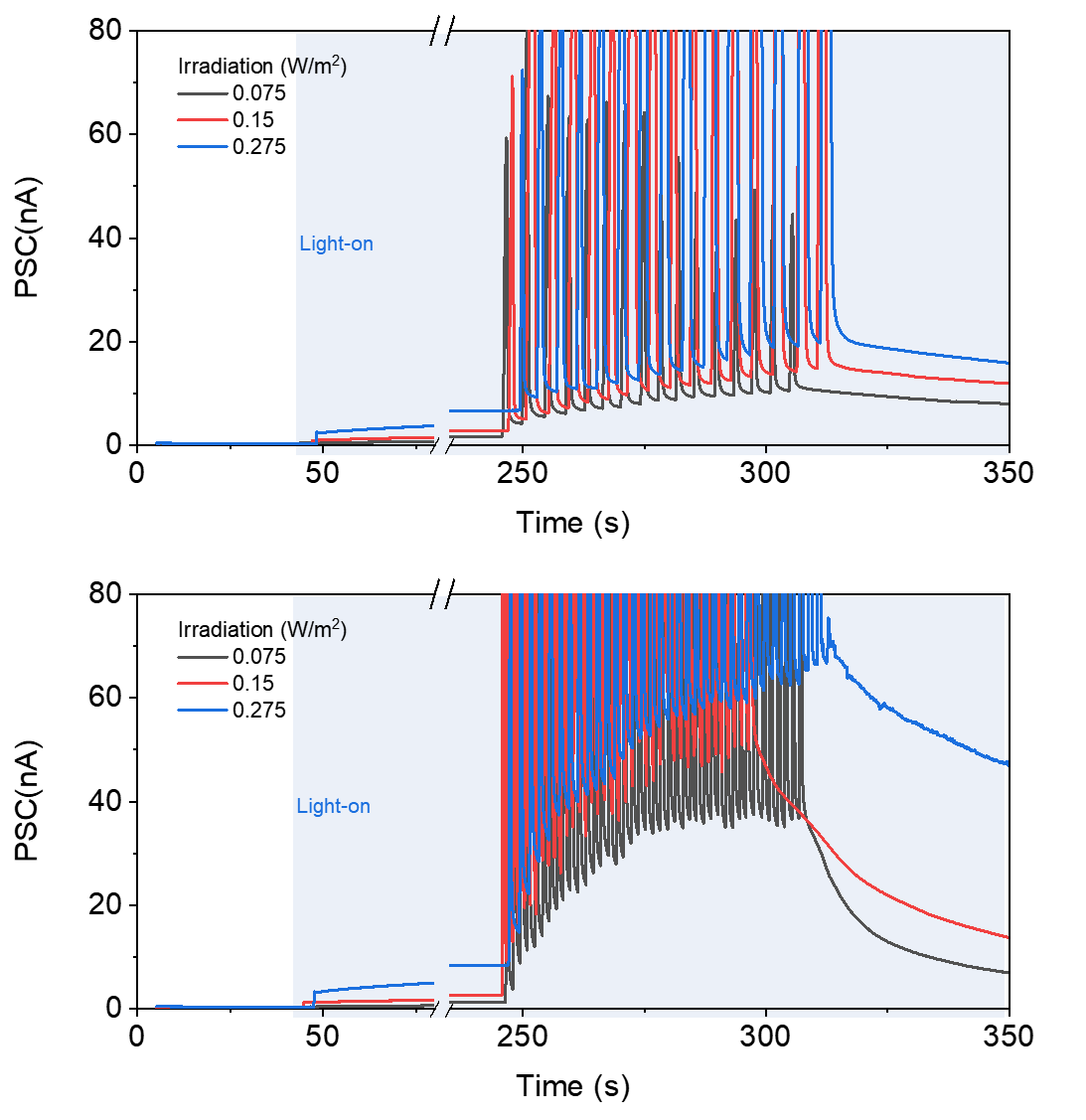


**Fig. S10 |** Real-time PSC for the BP2T synaptic sensor under various light irradiation and human breath inputs. Estimated respiratory rates of **a**, 15 and **b,** 25 breaths per minute.


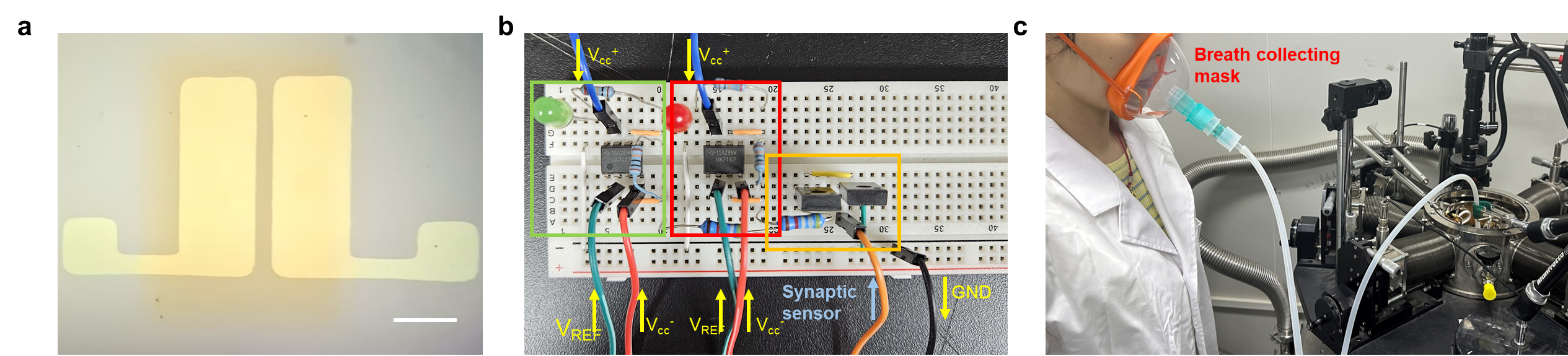


**Fig. S11 |** Overall configuration of measurement settings for healthcare device application. **a**, optical microscopic image of BP2T synaptic sensor. Scale bar, 200 μm. **b**, Amplifying and threshold-based computing circuit. **c**, Measurement settings for real-time breath collection following safety regulations.


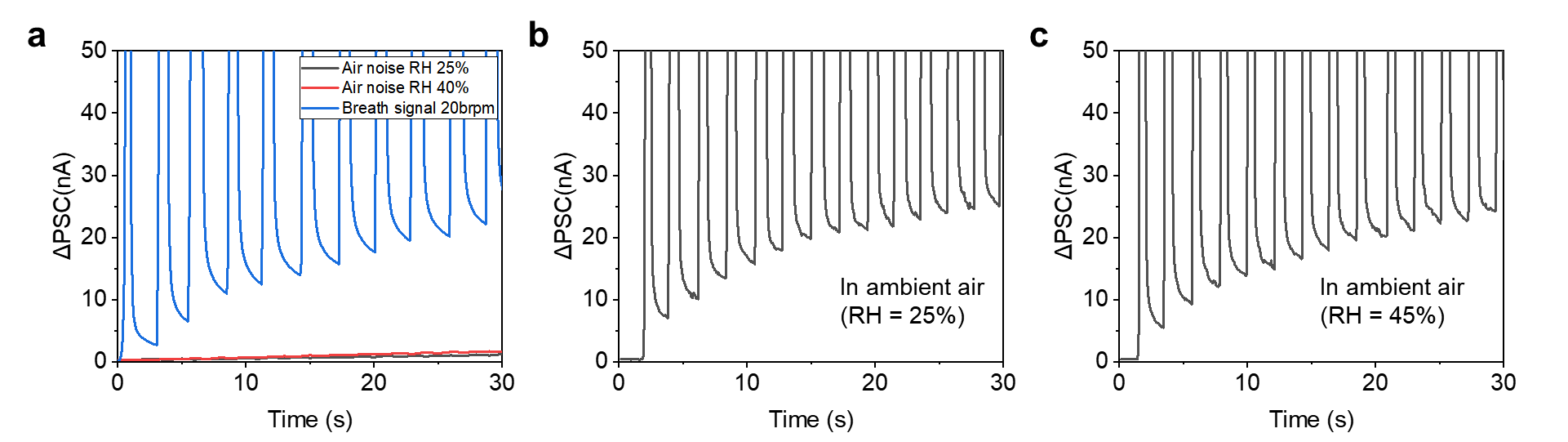


**Fig. S12 |** Real-time PSC plots of BP2T synaptic sensor under varied interferences. **a**, PSC change of synaptic sensor under various inputs including wind of ambient air with RH 25% (black), RH 45% (red), and human breath (blue). **b**, Real-time PSC plot upon breath inputs interfered by ambient air wind with RH 20% and **c**, RH 45%.
